# Is diagnostics of Benign Paroxysmal Positional Vertigo with a mechanical rotation chair superior to traditional manual diagnostics? A randomized controlled crossover study

**DOI:** 10.3389/fneur.2024.1519837

**Published:** 2024-12-06

**Authors:** Malene Hentze, Dan Dupont Hougaard, Herman Kingma

**Affiliations:** ^1^Balance and Dizziness Center, Department of Otorhinolaryngology, Head and Neck Surgery and Audiology, Aalborg University Hospital, Aalborg, Denmark; ^2^Department of Clinical Medicine, Aalborg University, Aalborg, Denmark

**Keywords:** vertigo, vestibular diseases, diagnostics, Benign Paroxysmal Positional Vertigo, BPPV, repositioning chair, mechanical rotation chair, TRV Chair

## Abstract

**Background:**

Benign Paroxysmal Positional Vertigo (BPPV) is the most common cause of vertigo. While various techniques and technologies have improved BPPV diagnostics and treatment, optimizing BPPV healthcare pathways requires a comprehensive understanding of the diagnostic modalities across diverse clinical settings.

**Objective:**

To compare traditional manual BPPV diagnostics (MD) with diagnostics done with the aid of a mechanical rotation chair (MRC) when using videonystagmography goggles with both modalities.

**Methods:**

This prospective, open-label, randomized diagnostic crossover study involved 215 adults with typical BPPV symptoms at a tertiary University Hospital-based outpatient clinic (Aalborg University Hospital, Denmark). Participants referred by general practitioners or otorhinolaryngologist clinics underwent both traditional manual and MRC diagnostics with the aid of videonystagmography goggles. The order of the diagnostic modalities was randomly assigned, and the two modalities were separated by a minimum of 30 min. The primary outcomes included sensitivity, specificity, positive predictive value (PPV), and negative predictive value (NPV) of traditional MD compared to MRC diagnostics. The secondary outcome was the agreement between the two modalities.

**Results:**

The MRC demonstrated a significantly higher sensitivity for BPPV detection in general for all participants (*p* = 0.00). Compared to MRC diagnostics, traditional MD displayed a sensitivity of 69.5% (95% confidence interval (CI): 59.8–78.1), specificity of 90.9% (95% CI: 83.9–95.6), PPV of 88.0% (95% CI: 83.9–95.6), and NPV of 75.8% (95% CI: 67.5–82.8). The overall inter-modality agreement was 80.5% (95% CI: 75.2–85.8, Cohen’s kappa 0.61). While both modalities detected unilateral posterior canal BPPV equally good (*p* = 0.51), traditional MD underperformed for non-posterior BPPV (significantly) and in subgroups referred by ENTs (trend) or with impaired cooperation during traditional MD (significantly).

**Conclusion:**

Traditional manual BPPV diagnostics remains a valuable first-line approach for most patients. However, MRC diagnostics offers advantages for complex BPPV cases, patients with impaired cooperation, patients referred from otorhinolaryngologist clinics, and those with negative traditional MD but an evident BPPV history. MRC may be useful as a second opinion diagnostic tool for treatment-resistant BPPV.

**Clinical trial registration:**

ClinicalTrials.gov identifier: NCT05846711.

## 1 Introduction

Benign Paroxysmal Positional Vertigo (BPPV) is the most common inner ear disorder that causes vertigo (1–4), accounting for up to 19% of all vertigo cases (5). By the age of 80, 10% of people will have experienced BPPV (3), with women being affected 1.5 to 2.0 times more often than men (3, 4, 6). The average onset of BPPV occurs between 43 to 57 years (3, 4, 6–8). While idiopathic BPPV is the most common, secondary BPPV (9–45%) can result from head trauma, vestibular neuritis, or other vestibular pathologies (4, 7).

The most accepted theory is that BPPV is caused by small calcium particles (otoconia) that detach from the maculae of the utricle and migrate into the semicircular canals (SCC), making them sensitive to gravity. The precise pathophysiological process underlying the otoconia detachment and displacement is not yet fully understood (2). The otoconia are most frequently dislodged in the posterior SCC (48–79%), followed by the lateral SCC (17–46%), and rarely the anterior SCC (1–3%) (4, 6, 8). Otoconia within a SCC can influence the cupular dynamics, causing BPPV, in two pathophysiological ways. The most frequent mechanism involves canalolithiasis, where the otoconia are free-floating within the canal’s endolymph. Head movements in the plane of the affected SCC cause gravity to exert a pulling force on these otoconia. This force, in turn, induces an endolymphatic flow and displaces the cupula. The cupular movement stimulates the sensory hair cells in the cupula and modifies the firing rate in the vestibular nerve, leading to the perception of a non-existent head rotation, expressed by the patient as vertigo (9). Cupulolithiasis represents a less common form of BPPV. In this scenario, the otoconia adhere directly to the cupula, increasing its density relative to the surrounding endolymph. By that, a head orientation change relative to the gravity vector results in a cupula deflection (10).

Both canalolithiasis and cupulolithiasis induce positional nystagmus and vertigo when the head orientation changes in the plane of the affected SCC (3, 4). The diagnosis of BPPV relies on the clinical assessment of the characteristic positional nystagmus and vertigo elicited by specific tests that rotate the patient’s head in the plane of the affected SCC. Traditional diagnostic tests, like the Dix-Hallpike Test for the posterior and anterior SCCs and the Supine Roll Test for the lateral SCC (11–13), are used for traditional manual bedside examination. The existing diagnostic criteria vary. The Bárány Society (12) and Japan Society for Equilibrium Research (13) require only a characteristic positional nystagmus during the diagnostic tests; the positional vertigo is only required in the patient’s history and not during the test. The American Academy of Otolaryngology, Head and Neck Surgery requires both objectifiable positional nystagmus and concomitant subjective vertigo to be present during the diagnostic tests (11).

Diagnostics of BPPV can be challenging for several reasons. One key challenge is that the presentation of BPPV varies greatly, and its symptoms and objective findings often overlap with those of other vestibular disorders (3, 5). This overlap makes it challenging to differentiate BPPV from other conditions, such as positional vertigo caused by vestibular migraine, central vestibular pathology (14), or conditions with changes in density between the cupula and endolymph (light cupula) not caused by dislocated otoconia (15). Light cupula is caused by unknown mechanisms or transiently by a substantial alcohol intake (16).

Finally, traditional manual diagnostics (MD) has limitations. These limitations include (1) variation of diagnostics between different examiners (inter-examiner variation) (17), (2) variation of diagnostics within the same examiner (intra-examiner variation), (3) variation in the interpretation of observed positional nystagmus, and (4) satisfactory degree of patient cooperation as a prerequisite (7). To ensure accurate diagnostics, it is essential to provide thorough instructions to the patient before starting the positional testing. In addition to explaining the need for rapid position changes, the patient should be instructed to keep their eyes wide open, blink as little as possible, and maintain a straight-ahead gaze direction. This instruction is necessary because the intensity and direction of positional nystagmus may vary with different gaze directions. When cases with posterior BPPV look toward the affected ear (the lower ear), the intensity of the torsional component increases, and the intensity of the vertical component in the ipsilateral eye decreases. In contrast, when cases with posterior BPPV look toward the non-affected ear (the upper ear), the torsional component decreases, and the vertical component of the nystagmus increases. If the examiner is unaware of these variations or fails to instruct the patient properly, it may lead to misinterpretation of the positional nystagmus (18).

The challenges in BPPV diagnostics can have serious consequences. Misdiagnosis or delays in treatment can lead to unnecessary medical interventions (3, 19), which is evident as 50% of patients with BPPV consult multiple medical subspecialties (3). Moreover, BPPV has a significant impact on daily life, with issues such as stopping driving (24%), social isolation (18%), and sick leave (37%) (3). This does not only affect daily life but also places a significant socioeconomic burden. The direct costs are estimated to be around $2000 per patient (20), and the total healthcare costs related to BPPV in the U.S. reach $2 billion annually (11).

Advances in BPPV diagnostics offer promising solutions to many of these challenges. Tools like videonystagmography (VNG) goggles and the mechanical rotation chair (MRC) are thought to improve accuracy, repeatability, and consistency in BPPV diagnostics and treatment. VNG goggles provide several benefits: (1) real-time visualization of the patient’s eye movements, (2) removal of visual fixation issues, (3) quantification of nystagmus patterns and characteristics (though most VNG systems are still limited tracking to horizontal and vertical eye movements), and (4) the ability to record the nystagmus. The MRC facilitates standardized, controlled, 360° multi-planar (yaw-, roll-, and pitch plane) head-on body movements, which allows the examiner to position the patient in precise positions aligned with the (assumed) anatomical locations of any of the six SCCs. This standardized test approach facilitates more reproducible and accurate test procedures and significantly reduces dependency on patient cooperation during clinical examinations and/or treatments. Previous research has shown promising results using these relatively new technologies (21–26). Bech et al. (7) found that diagnostics using the MRC in combination with VNG goggles were more sensitive for detecting BPPV than traditional MD with Frenzel glasses. However, the key question is whether the higher sensitivity was due to using an MRC, VNG goggles, or a combination of both (7).

A comprehensive understanding and optimization of diagnostic modalities across various clinical settings is essential to BPPV patient care. Given the widespread use of traditional MD with VNG goggles in otorhinolaryngologist (ENT) clinics and MRC diagnostics with VNG goggles in some highly specialized centers, this study aims to compare the BPPV diagnostic with these two diagnostic modalities. Employing a prospective, randomized, controlled crossover design, we will determine the sensitivity, specificity, positive predictive value (PPV), and negative predictive value (NPV) for traditional MD relative to MRC diagnostics. MRC diagnostics is the reference test, as a previous diagnostic study has shown this modality to be superior to traditional manual diagnostics (7). Additionally, we will assess the agreement between these diagnostic modalities for BPPV.

## 2 Materials and methods

### 2.1 Study design

The study employed an open-label randomized diagnostic crossover design. The design adhered to the Consolidated Standards of Reporting Trials (CONSORT) guidelines and incorporated principles from the Standards of Reporting of Diagnostic Accuracy Studies (STARD).

All participants underwent standardized procedures for BPPV diagnostics by two separate diagnostic modalities: traditional MD and MRC diagnostics. One examiner, who was trained and proficient in using both diagnostic modalities, performed all diagnostic procedures. Two experts in neurotology supervised the examiner before initiating the project and during the entire inclusion period. Randomization determined the initial diagnostic modality for the participant. The allocation ratio was 1:1, and randomization was done in permuted blocks of 4,6 and 8 (prepared with Sealed Envelope Ltd. 2022). To mitigate vertigo and nystagmus fatigue, we seated participants for at least 30 min before they crossed to the alternate diagnostic modality (27). Due to the nature of the interventions, blinding of participants or the examiner was not feasible.

### 2.2 Participants and setting

The study was conducted between April 12th, 2023, and January 11th, 2024, at a university hospital-based tertiary outpatient clinic. Potential participants were referred to the clinic by general practitioners in the North Denmark Region and ENTs in the North and Central Denmark Regions. General practitioners were instructed to refer participants presenting with a classic BPPV case history and to refrain from canalith repositioning maneuvers prior to referral. Conversely, potential participants referred by ENTs had, at the time of referral, already undergone one or several treatment attempts for BPPV with manual canalith repositioning maneuvers without successful relief of symptoms.

The examiner assessed the referred participants for eligibility. Inclusion criteria included age above 18 years of age, classic BPPV case history with short-lived (< 60 s) positional rotational vertigo with head changes relative to gravity, and sufficient written and spoken Danish proficiency to understand all aspects of the informed consent. Exclusion criteria included spontaneous- and/or gaze-evoked nystagmus, neck or spine immobility to a degree hindering traditional MD, physical limitations for MRC diagnostics (weight ≥ 150 kilos and or height ≥ 2 meters), insufficient cooperation during diagnostic testing, various medical conditions [heart failure (ejection fraction <40%), known cerebral aneurysm, recent cerebrovascular event (< 3 months) or arterial dissection disease], pregnancy, and intake of sedative antihistamines within the past 7 days. All participants eligible for inclusion were given oral and written information about the study, and their written consent was obtained before enrollment.

### 2.3 Materials

Traditional MD and MRC diagnostics were performed with the aid of VNG goggles using Video Frenzel goggles (VF405®, Interacoustics©, Middelfart, Denmark) with accompanying software (Micromedical VisualEyes™, version 3.1.0.203, Interacoustics©, Middelfart, Denmark). This allowed enlargement of eye images, video recording of eye movements during examinations, quantification and characterization of nystagmus parameters such as direction [vertical, horizontal, and/or torsional (only qualitatively)] and average slow phase velocities of vertical and horizontal nystagmus, if encountered. The MRC used in this study was the Thomas Richard-Vitton Repositional Chair (TRV Chair®, Interacoustics©, Middelfart, Denmark). An inertial measurement unit sensor (VORTEQ™, Interacoustics©, Middelfart, Denmark) was fixed to the goggles to secure visual feedback of the head positions during the MRC diagnostics.

BPPV was diagnosed and subcategorized according to the Bárány Society diagnostic criteria (12). Positional nystagmus was classified as specified in Table 1 and considered significant if the average slow phase velocity was ≥3 degrees/s and included ≥5 consecutive beats (28). BPPV was classified as primary or secondary BPPV. Secondary BPPV included past or present ipsilateral inner ear disease (excluding presbyacusis), previous ipsilateral middle- or inner ear surgery, or recent head trauma (<6 months before the onset of symptoms). In case of no evident etiology, the BPPV was classified as primary (idiopathic) BPPV.

**Table 1:** Positional nystagmus and BPPV classification.

		BPPV subtype	
		Canalolithiasis	Cupulolithiasis
BPPV Localization	Posterior semicircular canal	Ipsilateral Dix-Hallpike Test	
		Upbeating vertical nystagmus with a torsional component beating toward the affected side. Latency of one or a few seconds. Duration <1 min.	Upbeating vertical nystagmus with a torsional component beating toward the affected side. No latency. Duration >1 min.
	Lateral semicircular canal	Supine Roll Test	
		Bilateral geotropic horizontal nystagmus. No latency. Duration <1 min.	Bilateral apogeotropic horizontal nystagmus. No latency. Duration >1 min.
	Anterior semicircular canal	Contralateral Dix-Hallpike Test*	
		Downbeating vertical nystagmus ± torsional component beating toward the non-affected side. ± short latency. Duration <1 min.	Downbeating vertical nystagmus ± torsional component beating toward the non-affected side. ± short latency. Duration >1 min.

BPPV classification according to semicircular canal localization and subtype was based on characteristics of the positional nystagmus during relevant diagnostic positional testing (12).

^*^With anterior BPPV, the Dix-Hallpike Test may be positive bilaterally.

### 2.4 Interventions

All participants underwent screening for spontaneous- and gaze-evoked nystagmus and a vestibulo-ocular reflex function test, including a fixation-suppression test (manual rotation of the participant in the MRC with and without visual fixation). Participants with abnormal findings during these screenings were excluded from the study and received supplementary examinations following local clinical guidelines. Routinely, neither additional vestibular (no video head impulse test was performed to avoid potential displacements of otoliths) nor neurological examinations were included.

**Figure 1:**
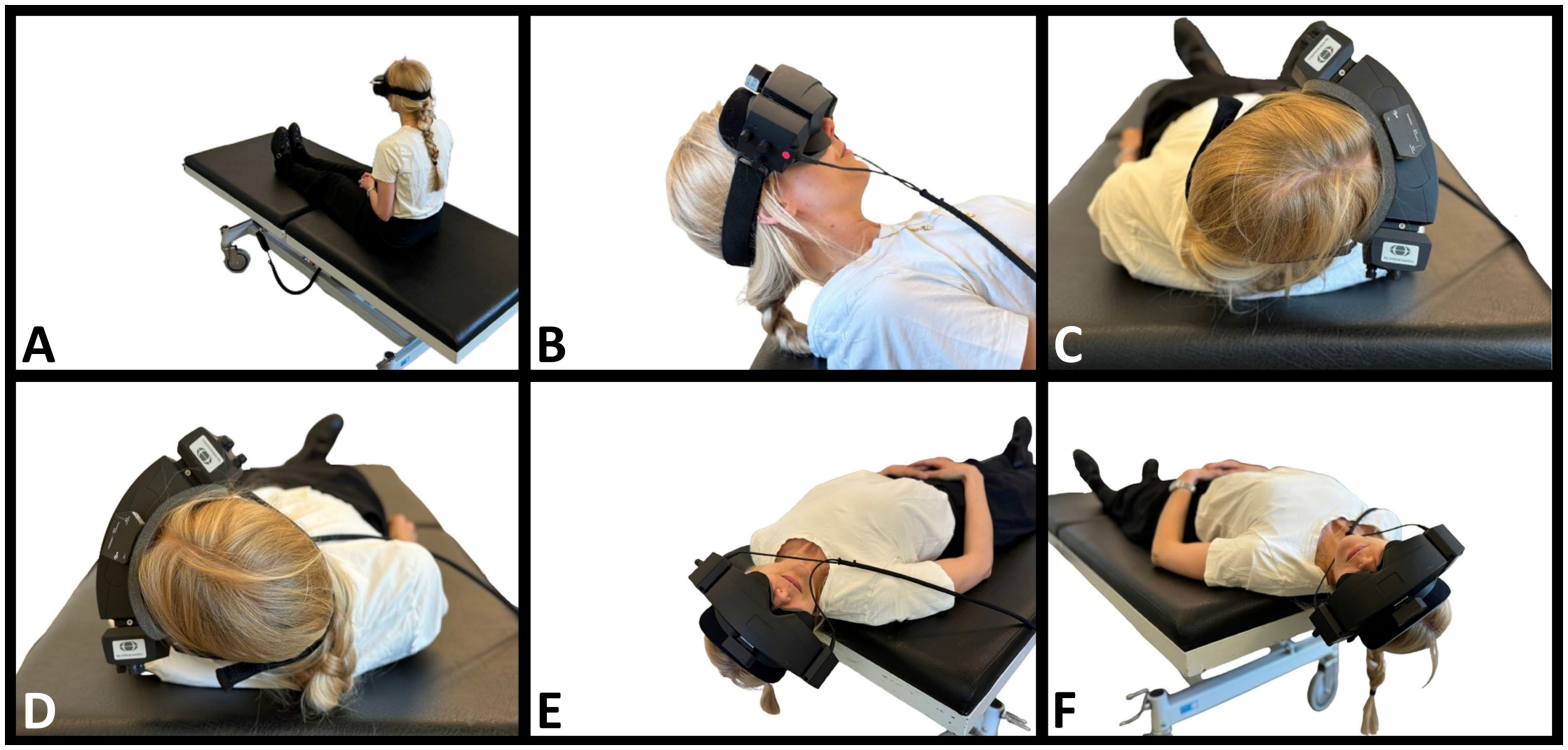
Traditional manual BPPV diagnostics. **(A)** The starting position. The participant is fitted with videonystagmography goggles and sits upright at the examination bed. **(B)** Supine position. Head flexed 30° to orientate the lateral semicircular canals in the vertical plane. **(C,D)** Right and left Supine Roll Test, respectively. From the supine position, the head is turned 90° to the right, then 180° to the left. **(E)** Right Dix-Hallpike Test. The head is turned 45° to the right, then moved backward to a supine position with the head extended to approximately 30° below the horizontal plane. **(F)** Left Dix-Hallpike Test. The head is turned 45° to the left, then moved backward to a supine position with the head extended to approximately 30° below the horizontal plane.

**Figure 2:**
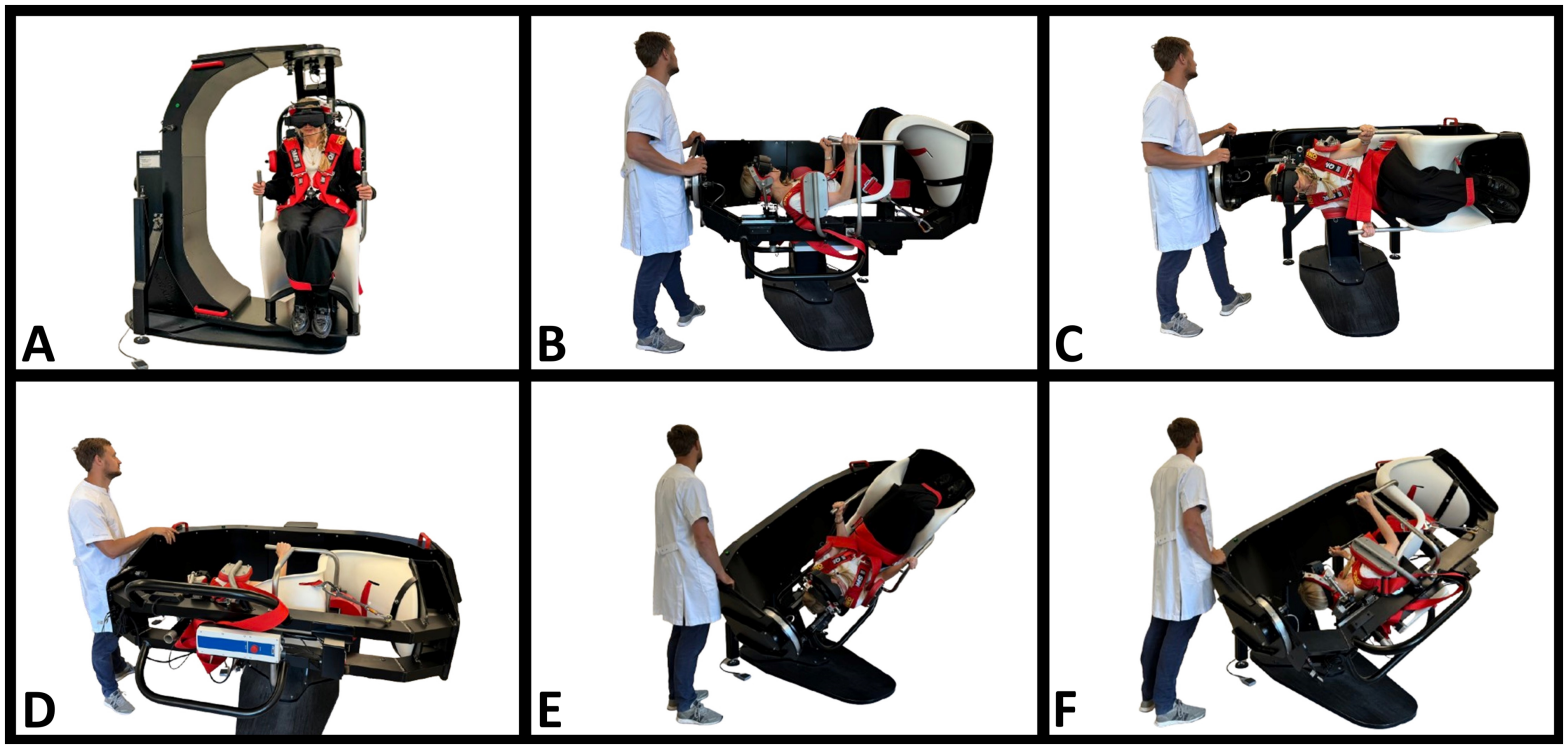
BPPV diagnostics with the mechanical rotation chair. **(A)** The starting position. The participant is fitted with videonystagmography goggles and seated in an upright position in the mechanical rotation chair (MRC). **(B)** Supine position. The participant’s neck is flexed approximately 30° using an integrated headrest. By this, the lateral semicircular canals are orientated in the vertical plane. **(C,D)** Right and left Supine Roll Test, respectively. From the supine position, the MRC is rotated 90° to the right in the roll plane of the MRC, then 180° to the left in the roll plane of the MRC. This results in head rotations around the lateral semicircular canals. **(E)** Right Dix-Hallpike Test. With the participant seated upright, the participant is rotated 45° to the right in the yaw plane of the MRC, followed by a 120° backward rotation in the pitch plane of the MRC. **(F)** Left Dix-Hallpike test. With the participant seated upright, the participant is rotated 45° to the left in the yaw plane of the MRC, followed by a 120° backward rotation in the pitch plane of the MRC.

For both diagnostic modalities, diagnostics were conducted in the same order (Figures 1, 2): Supine position (duration: 30 s), right Supine Roll Test, left Supine Roll Test (duration on each side: until nystagmus was recognized, or a maximum of 30 s), right Dix-Hallpike Test, and left Dix-Hallpike Test (duration on each side: 60 s). The Supine Roll Test was performed before the Dix-Hallpike Test to avoid that head movements in the plane of the posterior SCCs caused displacement of any otoconia in the lateral SCCs (29). To reduce the potential influence on the contralateral lateral SCC, the Supine Roll Test positions were completed as soon as any nystagmus was recognized and interpreted. The movement duration between positions was aimed to be identical for the two modalities (<2 s).

Also, the 3D head angulations were intended to be identical for both modalities in every position. These positions are described in Figure 1 (traditional MD) and Figure 2 (MRC diagnostics). As it was not possible to fixate the MRC at 120° backward in the pitch plane, we placed a physical mark on the MRC (indicating a 120° backward movement in the pitch plane), and real-time visual guidance was provided by the software and head movement sensor during MRC diagnostics. No visual guidance was provided during the traditional MD.

The video-recorded eye movements could be reviewed by the examiner on-site to support the diagnostic conclusion. A blinded expert later reviewed all recorded videos of eye movements during diagnostics. The expert assessment was then compared to the examiner’s diagnostic conclusion for each participant to validate the on-site diagnostic conclusion. All diagnostic conclusions presented in this paper reflect the original diagnosis of the examiner.

The examiner determined the degree of participant cooperation during traditional MD and classified them into the following three groups: sufficient, impaired but acceptable, and insufficient. Sufficient cooperation was defined as reaching the aimed head angulation in 3D for both the supine roll and Dix-Hallpike Tests. Impaired but acceptable cooperation was defined as a Supine Roll Test with rotations in the yaw plan of a minimum of 2/3 of the aimed 90°, Dix-Hallpike Test with rotations in the yaw plane of a minimum of 2/3 of the aimed 45° or rotation in the pitch plane of minimum 100°. Insufficient cooperation was defined as rotations that were less in terms of angulation than what was defined as impaired but acceptable cooperation. In case of insufficient cooperation, the participants were excluded from the study and offered MRC diagnostics outside the study protocol.

All participants diagnosed with BPPV were offered subsequent targeted treatment with the MRC.

### 2.5 Power calculation

*A priori* power calculation was conducted with an independently certified biostatistician who provided statistical advice throughout the study. Based on a contingency table for reporting diagnostic data (30), 203 participants were required to achieve a diagnostic sensitivity of 90% with a confidence interval of 5% (on each side). Based on existing literature describing the same population, a prevalence of BPPV of 68% among the referred participants was anticipated (7).

### 2.6 Data collection

All data were collected at one site (the Balance and Dizziness Centre, Department of Otorhinolaryngology, Head and Neck Surgery and Audiology, Aalborg University Hospital, Denmark). Data collection was performed by the same non-blinded examiner and included history taking, physical examination, and electronic patient record review. Data was managed using REDCap® (Research Electronic Data Capture) version 13.1.37 hosted at a secure server in the North Denmark Region (31, 32).

### 2.7 Statistical analysis

All data analyses were per-protocol analyses and included only participants who completed the trial. Due to the predefined index and reference test, analysis was not performed blinded to randomization.

Descriptive statistics were used to summarize baseline characteristics. Continuous variables were reported with mean and standard deviation (or median and range in variables with skewed distribution). Normality was checked visually and with the Kolmogorov-Smirnoff test. Categorical variables were reported with absolute and relative frequencies.

For comparison between two unpaired groups, Student’s t-test (continuous data) and Chi-square (Fisher’s exact test in case of expected <5 in a cell) (categorical data) were used. Comparison between two paired groups was only performed on categorical data using McNemar’s test (McNemar’s exact test in case of expected <5 in a cell). The primary outcome measure was sensitivity, specificity, PPV, and NPV, including 95% confidence intervals (CI) of traditional manual BPPV diagnostics (index test) compared to MRC diagnostics (reference test). The reference and index tests were chosen based on the literature (7). The secondary outcome was agreement between the two diagnostic modalities, evaluated through an overall agreement percentage and Cohen’s kappa (33).

During analysis, the population was divided into two groups: posterior BPPV (the most frequent localization of BPPV) and non-posterior BPPV. Non-posterior BPPV was used to classify all lateral, anterior, and multicanal (involvement of >1 SCC) BPPV. Analyses were evaluated with significance set at alpha level 0.05. All statistical analyses were performed using Stata/MP version 18.

## 3 Results

**Figure 3:**
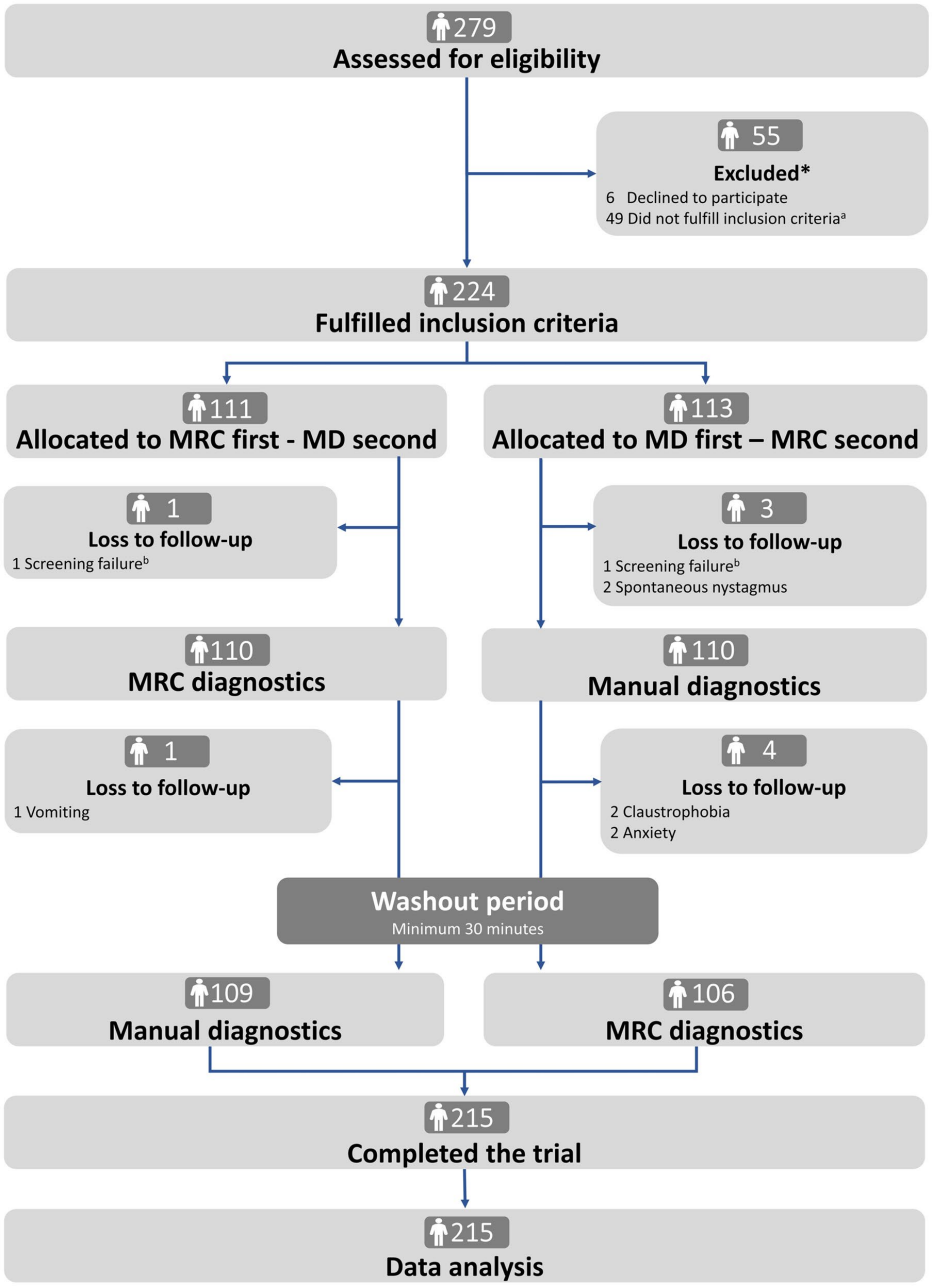
Trial profile. MRC: mechanical rotation chair; MD: manual diagnostics. *There was no difference in age and sex between excluded and included participants (Supplementary Table A). ^a^The reasons for not fulfilling the inclusion criteria were remission of symptoms (*n* = 34), spontaneous- and gaze-evoked nystagmus (*n* = 1), did not understand Danish (*n* = 2), pregnant (*n* = 1), neck- and back immobility (*n* = 5), sedative antihistamines (*n* = 2), and comorbidities (*n* = 4). ^b^Screening failure included intake of sedative antihistamines and recent cerebral hemorrhage (<3 months).

Of the 279 participants screened, 55 (19.7%) were excluded, mainly due to spontaneous remission of symptoms (34/55, 61.8%). Two hundred twenty-four participants met the inclusion criteria and were randomly assigned to traditional MD or MRC diagnostics as the first test modality. A total of nine participants (9/224, 4.0%) withdrew (*n* = 5, vomiting, claustrophobia, or anxiety) or were excluded (*n* = 4, screening failure or spontaneous nystagmus) after randomization, leaving 215 participants who completed the study (*n* = 109 and *n* = 106 in the two randomization groups). No participants reported any serious adverse events (Patient flow is illustrated in Figure 3).

Baseline characteristics (Table 2) showed a predominance of women (68.9%) with a mean age of 58.1 years (ranging from 18 to 91 years). The majority were referred by general practitioners (85.6%), with a BPPV confirmation rate of 50.0%. The confirmation rate was 74.2% for participants referred by ENTs. Primary BPPV was more common (83.5%) than secondary BPPV (16.5%). No differences were observed in the baseline variables according to the randomized groups.

**Table 2:** Demographics and BPPV characteristics (*n* = 215).

Demographics	Total (*n* = 215)		MRC-MD (*n* = 109)		MD-MRC (*n* = 106)		*p*-value
Female, *n* (%)	148	(68.9)	75	(68.8)	73	(68.9)	0.99
Age, mean years ±SD	58.1	±15.9	56.7	±14.5	59.6	±17.1	0.18
Age, years [range]		[18–91]		[22–82]		[18–91]	
Duration of symptoms, days, median [range]	36	[2–1829]	41	[3–1829]	34	[2–1,095]	0.71
Referred by:							
General practitioner, *n* (%)	184	(85.6)	96	(88.1)	88	(83.0)	0.29^a^
ENT clinics, *n* (%)	31	(14.4)	13	(11.9)	18	(17.0)	0.29^a^
Degree of cooperation during traditional MD:							
Sufficient cooperation, *n* (%)	175	(81.4)	93	(85.3)	82	(77.4)	0.13^a^
Impaired, but acceptable cooperation, *n* (%)	40	(18.6)	16	(14.7)	24	(22.6)	0.13^a^
BPPV characteristics							
Confirmed BPPV total^†^, *n* (%)	115	(53.5)	53	(48.6)	62	(58.5)	0.14
Confirmed BPPV if referred by a general practitioner^†^, *n* (%)	92	(50.0)	44	(45.8)	48	(54.5)	0.24
Confirmed BPPV if referred by an ENT clinic^†^, *n* (%)	23	(74.2)	9	(69.2)	14	(77.8)	0.59
Primary BPPV, *n* (%)	96	(83.5)	44	(83.0)	52	(83.9)	0.90^a^
Secondary BPPV, *n* (%)	19	(16.5)	9	(17.0)	10	(16.1)	0.90^a^
Vestibular neuritis, *n* (%)	8	(7.0)	3	(5.7)	5	(8.1)	0.72
Ipsilateral sudden deafness, *n* (%)	1	(0.9)	1	(1.9)	0	(0.0)	0.46
Meniere’s disease, *n* (%)	0	(0.0)	0	(0.0)	0	(0.0)	–
Ipsilateral ear surgery, *n* (%)	2	(1.7)	1	(1.9)	1	(1.6)	–
Head trauma less than 6 months before the onset of vertigo, *n* (%)	8	(7.0)	4	(7.6)	4	(6.5)	–

MRC, mechanical rotation chair; MD, manual diagnostics; ENT, otorhinolaryngologist.

The randomized groups sort the table: MRC-MD (MRC diagnostics first and MD second) and MD-MRC (MD first and MRC diagnostics second).

^†Confirmed BPPV with MD and/or MRC diagnostics.^

^aPairwise dependent p-values.^

No demographics or BPPV characteristics differ significantly between the two groups (all *p*-values > 0.05).

The MRC diagnostics had a significantly higher sensitivity in detecting BPPV than traditional MD (*p* = 0.00) (Table 3). Of the BPPVs diagnosed with the MRC, 81.0% were categorized as single canal primarily in the posterior SCC (63.8%), followed by the lateral SCC (16.2%) and anterior SCC (1.0%). The remaining 19.1% of the cases with BPPV involved more than one SCC: 10.5% with bilateral posterior BPPV and 8.6% with ipsilateral combined affection of the posterior and lateral SCC. MRC diagnostics identified a significantly higher proportion of non-posterior (*p* = 0.00) and unilateral multicanal (*p* = 0.01) BPPV than traditional MD. Both diagnostic modalities detected an overall higher proportion of right-sided BPPV (Supplementary Table B), but the detection of left-sided BPPVs was better for the MRC diagnostics compared to traditional MD (*p* = 0.02) (Table 3).

**Table 3:** BPPV characteristics (*n* = 215).

	MRC		MD		
	*n*	(%)	*n*	(%)	*p*-value
BPPV, total	105	(48.8)	82	(38.6)	0.00*
	*n* = 105		*n* = 83		
BPPV laterality:					
Right	53	(50.5)	47	(56.6)	0.16
Left	41	(39.1)	29	(34.9)	0.02*
Bilateral	11	(10.5)	7	(8.4)	0.22
BPPV localization and subtype:					
Single canal BPPV	85	(81.0)	74	(89.2)	0.12
Posterior BPPV total	67	(63.8)	63	(75.9)	0.51
Posterior CAN	61	(58.1)	59	(71.1)	0.74
Posterior CUP	6	(5.7)	4	(4.8)	0.32
Non-posterior BPPV	38	(36.2)	20	(24.1)	0.00*
Lateral BPPV total	17	(16.2)	9	(10.8)	0.06
Lateral CAN	4	(3.8)	3	(3.6)	–
Lateral CUP	13	(12.4)	6	(7.2)	0.07
Anterior BPPV total	1	(1.0)	2	(2.4)	–
Anterior CAN	1	(1.0)	1	(1.2)	–
Anterior CUP	0	(0.0)	1	(1.2)	–
Multicanal BPPV (> 1 SCC):	20	(19.1)	9	(10.8)	0.01*
Unilateral multicanal	9	(8.6)	2	(2.4)	0.04
Bilateral single canal	11	(10.5)	6	(7.2)	0.13
Bilateral multicanal^†^	0	(0.0)	1	(1.2)	–

MRC, mechanical rotation chair; MD, traditional manual diagnostics; CAN, canalolithiasis; CUP, cupulolithiasis; SCC, semicircular canal.

^†Bilateral multicanal: Bilateral BPPV with minimum one laterality involving ≥ 2 SCC.^

Significant *p*-values (*p*-value ≤ 0.05) are marked with an asterisk. Please note that diagnostics with an MRC were more sensitive in detecting BPPV and found a significantly higher proportion of non-posterior and multicanal BPPV. Both modalities detected a higher proportion of right-sided BPPV. All positional tests used in this study began by examining the right side.

In approximately 80% of participants, the two diagnostic modalities agreed on whether to confirm or rule out BPPV (Table 4). In nearly 20% of cases (19.5%), the modalities disagreed, meaning that only one diagnostic modality identified BPPV, with the MRC diagnostics detecting more BPPV cases (14.9%) than traditional MD (4.4%). Traditional MD missed a considerable number of non-posterior BPPV cases. However, when focusing specifically on posterior BPPV, there was no significant difference between the two modalities in their ability to confirm or rule out the diagnosis of posterior canal BPPV (*p* = 0.51).

**Table 4:** Results of BPPV diagnostics (*n* = 215).

All BPPV						
		MRC			*p*-value	
		BPPV	No BPPV	Total	0.00*	
MD	BPPV	73	10	83 (38.6)		
	No BPPV	32	100	132 (61.4)	Accuracy of MD	(95% CI)
	Total	105 (48.8)	110 (51.2)	215 (100.0)	80.5%	(75.2–85.8)

Posterior BPPV						
		MRC			*p*-value	
		P BPPV	Non-P BPPV or no BPPV	Total	0.51	
MD	P BPPV	47	16	63 (29.3)		
	Non-P BPPV or no BPPV	20	132	152 (61.4)	Accuracy of MD	(95% CI)
	Total	67 (31.2)	148 (68.8)	215 (100)	83.3%	(78.3–88.3)

Non-posterior BPPV						
		MRC			*p*-value	
		Non-P BPPV	P BPPV or no BPPV	Total	0.00*	
MD	Non-P BPPV	16	4	20 (9.3)		
	P BPPV or no BPPV	22	173	195 (90.7)	Accuracy of MD	(95% CI)
	Total	38 (17.7)	177 (82.3)	215 (100)	87.9%	(83.6–92.3)

MRC, mechanical rotation chair; MD, traditional manual diagnostics; P, posterior.

Results are expressed as *n* (%). Percentages refer to the total sample within each group.

Significant *p*-values (*p*-value ≤ 0.05) are marked with an asterisk.

Please note that diagnostics with an MRC was the most sensitive test modality with BPPV diagnostics in all groups except those with posterior BPPV.

Table 5 shows the diagnostic performance of traditional MD relative to the MRC diagnostics. For traditional MD, the sensitivity was 69.5% (95% CI: 59.8–78.1), the specificity was 90.9% (95% CI: 83.9–95.6), the PPV was 88.0% (95% CI: 83.9–95.6), and the NPV was 75.8% (95% CI: 67.5–82.8). For non-posterior BPPV, the sensitivity significantly lowered to 42.1% (95% CI: 26.3–59.2). However, the specificity remained high (97.7, 95% CI: 94.3–99.4) and the NPV was significantly higher [94.7% (95% CI: 83.4–92.8)]. Additionally, the data indicated a trend toward lower sensitivity of traditional MD for participants referred by ENTs and those with reduced cooperation during the examination.

**Table 5:** Diagnostic profile of traditional manual diagnostics (*n* = 215).

	Traditional MD compared to mechanical rotation chair diagnostics							
	Sensitivity		Specificity		PPV		NPV	
	%	(95% CI)	%	(95% CI)	%	(95% CI)	%	(95% CI)
BPPV total	69.5	(59.8–78.1)	90.9	(83.9–95.6)	88.0	(79.0–94.1)	75.8	(67.5–82.8)
Posterior BPPV	70.1	(57.7–80.7)	89.2	(83.0–93.7)	74.6	(62.1–84.7)	86.8	(80.4–91.8)
Non-posterior BPPV	42.1	(26.3–59.2)	97.7	(94.3–99.4)	80.0	(56.3–94.3)	88.7	(83.4–92.8)
Referred by:								
General practitioner (*n* = 184)	70.9	(60.1–80.2)	93.9	(87.1–97.7)	91.0	(81.5–96.6)	78.6	(70.1–85.7)
ENT clinics (*n* = 31)	63.2	(38.4–83.7)	66.7	(34.9–90.1)	75.0	(47.6–92.7)	53.3	(26.6–78.7)
Cooperation during MD:								
Sufficient cooperation (*n* = 175)	75.3	(64.2–84.4)	92.9	(85.8–97.1)	89.2	(79.1–95.6)	82.7	(74.3–89.3)
Impaired but acceptable cooperation (*n* = 40)	53.6	(33.9–72.5)	75.0	(42.8–94.5)	83.3	(58.6–96.4)	40.9	(20.7–63.6)
MD performed:								
First (*n* = 106)	64.9	(51.1–77.1)	89.8	(77.8–96.6)	88.1	(74.4–96.0)	68.8	(55.9–79.8)
Second (*n* = 109)	75.0	(60.4–86.4)	91.8	(81.9–97.3)	87.8	(73.8–95.9)	82.4	(71.2–90.5)

MD, traditional manual diagnostics (index test); MRC, mechanical rotation chair (reference test); PPV, positive predictive value; NPV, negative predictive value; ENT, otorhinolaryngologist.

Non-posterior BPPV: lateral, anterior, and multicanal BPPV.

Please note the significance of the higher specificity and NPV and lower sensitivity of traditional MD with diagnostics of non-posterior BPPV compared to overall BPPV diagnostics.

The overall agreement between traditional MD and MRC diagnostics was good, with an agreement rate of 80.5% (95% CI: 75.2–85.8) and a Cohen’s kappa of 0.61 (moderate agreement) (Table 6). There was no change in agreement or Cohen’s kappa in cases with posterior BPPV. However, for cases with non-posterior BPPV, there was a trend of a higher agreement but a lower Cohen’s kappa.

**Table 6:** Agreement between the diagnostic modalities (*n* = 215).

	Overall agreement		Cohen’s kappa	
	%	(95% CI)		(95% CI)
BPPV or no BPPV	80.5	(75.2–85.8)	0.61	(0.48–0.74)
Agreement on subtype and localization	79.0	(73.4–84.6)	0.57	(43.1–70.1)
Posterior BPPV	83.3	(78.3–88.2)	0.60	(0.47–0.74)
Non-posterior BPPV	87.9	(83.5–92.3)	0.49	(0.36–0.61)
Referred by:				
General practitioner (*n* = 184)	83.2	(77.7–88.6)	0.66	(0.52–0.80)
ENT clinics (*n* = 31)	64.5	(47.7–81.4)	0.29	(−0.06–0.63)
Cooperation during MD:				
Sufficient cooperation (*n* = 175)	85.1	(78.9–90.4)	0.69	(0.55–0.84)
Impaired but acceptable cooperation (*n* = 40)	60.0	(44.8–75.2)	0.23	(−0.04–0.50)
MD performed:				
First (*n* = 106)	76.4	(68.3–84.5)	0.54	(0.35–0.72)
Second (*n* = 109)	84.4	(77.6–91.2)	0.68	(0.49–0.86)

MRC, mechanical rotation chair; MD, traditional manual diagnostics; ENT, otorhinolaryngologist.

Non-posterior BPPV: lateral, anterior, and multicanal BPPV.

Please note that Cohen’s kappa is significantly lower in cases referred by ENTs and with impaired cooperation during MD. All p-values for Cohen’s kappa are ≤ 0.05, indicating that the agreement between the modalities is beyond chance.

The agreement between the two modalities seemed to be influenced by several factors. Participants with impaired cooperation during traditional MD showed significantly lower agreement and Cohen’s kappa values. Similarly, lower agreement and Cohen’s kappa were observed in participants referred by ENTs, although this was not statistically significant.

When the two randomized groups were compared, traditional MD showed a trend toward a higher sensitivity when it was performed as the second test modality (Table 5). The agreement and Cohen’s kappa were also higher in this subgroup (Table 6).

## 4 Discussion

### 4.1 Key findings

This study compared traditional MD with the more advanced MRC diagnostics (both modalities with the use of VNG goggles). While both modalities effectively identified BPPV, the MRC diagnostics demonstrated significantly higher sensitivity than traditional MD. When the MRC diagnostics was considered as the reference standard, traditional MD correctly identified approximately 70% of BPPV-positive cases (sensitivity) and 90% of BPPV-negative cases (specificity) (Table 5). Around 90% of the positive results from traditional MD were correct (PPV). However, false negative results with traditional MD occurred in about 25% of cases (NPV), suggesting that a negative result from traditional MD does not necessarily rule out BPPV.

For non-posterior BPPV, traditional MD showed a significantly lower sensitivity. However, its ability to correctly identify the negative cases (NPV) improved for these cases. Traditional MD also proved less accurate in more complex cases, such as participants referred by ENTs with retractable BPPV and/or atypical BPPV cases and in those with a low level of cooperation during the diagnostics (Table 5). However, the PPV and NPV from subgroups where the prevalence of BPPV was low, such as non-posterior BPPV, should be interpreted with caution as the predictive measures are influenced by the prevalence (34).

In the majority of participants (80%), traditional MD and MRC diagnostics agreed on whether the participant had BPPV or not. This agreement was confirmed by Cohen’s kappa coefficient of 0.61 (moderate agreement) (33). The level of agreement varied depending on the subgroup analyzed, as it was lower in groups with non-posterior BPPV (weak agreement), participants referred by ENTs (minimal agreement), and with compromised cooperation during the examination (minimal agreement) (33) (Table 6). While Cohen’s kappa is useful for adjusting for chance agreement, it is important to underline that it can be influenced by the prevalence of BPPV (35). A paradox occurs where high agreement in subgroups with low BPPV prevalence results in a low kappa value (35). This paradox might be present in data from participants referred by ENTs and in participants with impaired cooperation, which both had unbalanced data distributions in the contingency tables (Supplementary Table D).

In about 20% of the cases, the two diagnostic modalities had conflicting diagnostic conclusions. BPPV was exclusively detected with the MRC in roughly 90% of these cases. Conversely, traditional MD occasionally identified BPPV cases that were not confirmed by the MRC. It is complex to interpret the cases that had a positive BPPV result with traditional MD but a negative BPPV result with MRC diagnostics. Either they represent ‘true false positives’ where the traditional MD wrongly concluded a positive BPPV, or they represent cases with true BPPV that were only detected with traditional MD but missed with the MRC diagnostics – in this case, falsely lowering the specificity and PPV for traditional MD when compared to the MRD diagnostics. This highlights a fundamental challenge in diagnostic studies: the designation of a reference test. By definition, a reference test is assumed to be the most accurate test available. However, it carries an unknown level of uncertainty that cannot be adjusted in the analysis.

Consequently, any comparison test, in this case, traditional MD, will perform worse. However, our choice of the MRC diagnostics as the reference test is supported when looking at the overall superior sensitivity of the MRC diagnostics compared to traditional MD (Table 4). This choice is further reinforced when considering the supplementary analysis that demonstrates the performance of the MRC diagnostics when considered as the index test compared to traditional MD as the reference test (Supplementary Table G).

### 4.2 Comparison with existing literature

There is limited research that compares traditional MD with MRC diagnostics. Our study is the first to compare these modalities with the concomitant use of VNG goggles for both modalities. Bech et al. (7) previously found that BPPV diagnostics with MRC and VNG goggles are more sensitive than traditional MD with Frenzel glasses. However, in our study, adding VNG goggles to traditional MD did not significantly improve the sensitivity and NPV of traditional MD (compared to MRC diagnostics) or the overall agreement (and kappa coefficient) between the two modalities when compared to the findings by Bech et al. (7). This finding was also evident in challenging subgroups, such as participants referred by ENTs and those with reduced cooperation. However, we observed a trend toward better agreement for non-posterior BPPV when VNG goggles were added to the traditional MD in our study. Given the similarity between our results and those reported by Bech et al. (7), the superior performance of MRC diagnostics might be caused by the benefits of the MRC itself rather than the added value of concomitant usage of VNG goggles.

Previous studies have suggested that the use of VNG goggles during BPPV diagnostics might lead to overdiagnosis of BPPV as non-pathological positional nystagmus in healthy individuals could be misinterpreted during the positional testing (21, 36, 37). However, our findings did not support this concern, as we found a similar BPPV detection rate (54%) with VNG goggles compared to the detection rate (57%) with Frenzel glasses in Bech et al. (7). On the contrary, it could be hypothesized that VNG goggles may reduce the risk of overdiagnosis in case of non-pathological positional nystagmus, as the appertaining software helps the examiner in determining direction(s) of any observed positional nystagmus. Moreover, a less experienced examiner might be more likely to diagnose BPPV based exclusively on the presence of any positional nystagmus. Nonetheless, the real risk of BPPV overdiagnosis could be the VNG goggles’ ability to detect nystagmus with very low slow phase velocities.

We found the lack of improvement of sensitivity with traditional MD and concomitant use of VNG goggles unexpected. One possible explanation is that the examiners in Bech et al.’s study (7) were more experienced in the interpretation of positional nystagmus than the examiner in this study. The VNG goggles might have compensated for this difference in experience, leading to an underestimation of their potential benefits. However, we found no significant difference in BPPV detection rates throughout the study period (Supplementary Table G), which suggests that a possible learning curve of the examiner in our study did not influence the interpretation of positional nystagmus.

There may exist alternative explanations for the limited diagnostic superiority of VNG goggles in comparison to Frenzel glasses. First, while VNG goggles may suppress visual fixation more effectively, this might have minimal impact in identifying eye movements specific to BPPV, as rotatory eye movements are typically unaffected by visual fixation (18). Second, it might be more difficult to detect eye rotation with black-and-white infrared VNG images compared to Frenzel glasses that provide a full-color image of the scleral arteries and iris to better identify eye rotation for BPPV diagnostics; sacrificing may be the advantage of less fixation suppression as when using infrared VNG. Comparative studies could and should clarify these issues.

Our results showed that only 64% of the participants referred by general practitioners had the most common type (single canal posterior) of BPPV when diagnosed with the MRC. The remainder of the participants had non-posterior or multicanal involvement (Table 3). Traditional MD missed a significant portion of these non-posterior and multicanal BPPVs, which is consistent with previous research (7, 21, 23, 24). Hereby, BPPV diagnostics with the MRC seem to detect a broader range of BPPV presentations than traditional MD.

However, studies have reported different frequencies of the SCC localizations of BPPV. With traditional MD, the reported frequency of lateral BPPV is 8–46%, and multicanal BPPV is 2–12% (8, 38–40). This variance between studies suggests that non-posterior BPPV might be underdiagnosed in some clinics. The potential underdiagnosed non-posterior BPPV might be due to differences in diagnostic protocols or adherence to them. A retrospective study by Lloyd et al. (41) showed that the majority of patients who undergo BPPV diagnostics are only tested for posterior BPPV and not lateral BPPV. Also, Bhandari et al. (8) proposed, supported by 3D simulation models (29), that the sequence of positional tests might influence diagnostic results. In theory, the Dix-Hallpike Test, which is predominantly the initial test in cases where BPPV is suspected (11), displaces otoconia present in the lateral SCC(s), thereby potentially affecting the outcome of the Supine Roll Test and, theoretically, causes BPPV to be underdiagnosed. However, we could not reproduce the high frequency of lateral BPPV seen by Bhandari et al. (8) despite performing the Supine Roll Test prior to the Dix-Hallpike Test.

Our study found a higher prevalence of right-sided BPPV (Table 3; Supplementary Table B), which aligns with previous studies (39, 42). This could be due to our testing protocol first starting all positional tests on the right side. Additionally, we found a significantly higher rate of right-sided BPPVs with traditional MD than MRC diagnostics (Supplementary Table B), suggesting that traditional MD might be more affected by the order of tests. Unfortunately, many studies do not report the starting side of the positional tests, making it difficult to evaluate if the higher rate of right-sided BPPVs is caused by the order of individual tests and choice of initial side (8, 39, 42).

When comparing BPPV studies, it is of paramount importance to consider any population differences. The majority of patients with BPPV are diagnosed and treated in primary care (3), while research is often conducted in highly specialized hospital-based centers. Our participants were referred by general practitioners, with only around 15% referred by ENTs. We observed differences in BPPV diagnoses and the distribution of BPPV types between these groups (Table 2; Supplementary Table E), suggesting that patients referred to specialized centers (by ENT clinics) are more complex BPPV cases than patients referred to ENTs (by general practitioners). However, these findings differ from previous studies with a similar population (7, 23). The reasons for these differences are unclear, but they might be related to specific study protocols.

Finally, this study confirmed that reduced patient cooperation negatively affects traditional MD, as reported previously (7). This limitation is addressed by MRC diagnostics, which can be performed regardless of a patient’s ability to physically cooperate, allowing for examinations in patients with, e.g., neck and back immobility. Another benefit of the MRC is the standardization of the procedures related to the diagnostics and treatment of BPPV (the MRC used in this study has many pre-set positions), supposedly reducing inter- and intra-examiner variability. The MRC used in this study simplifies these procedures due to fixed positioning in the yaw, roll, and pitch axes. However, these fixed intervals might disadvantage patients with atypical inner ear anatomy (43). Ideally, an MRC device for such patients would allow for individualized angles, like the Rotundum® Rotary Chair (Balcare GmbH, Küsnacht, Switzerland). Still, angulation velocity and interval duration are not yet standardized and need further study.

### 4.3 Strengths and limitations

This prospective, block-randomized crossover study was carefully designed to minimize bias. The same examiner thoroughly performed all examinations with the two diagnostic modalities. This ensured consistency with the diagnostic procedures and the interpretation of findings, eliminating bias due to inter-examiner variation. To ensure high-quality BPPV diagnostics, the examiner underwent thorough training before initiating the data collection and maintained consistent application of the modalities throughout the study period. This consistency is supported by the finding of no difference in the BPPV detection rates between the first and second study periods, indicating no learning curve effect (Supplementary Table H). Among referred participants, 20% (55/279) were ineligible for inclusion, and there was no selection bias concerning age and sex (Supplementary Table A). Of the included participants, there was no significant difference in demographics, referral pattern, and BPPV etiology between the two randomized groups (Table 2), and the randomization process was concluded sufficient. The study achieved a high completion rate (215/224 participants) with only 4% (9/224) dropout (Figure 3). The majority of participants did not experience any side effects or expressed any major complaints during the BPPV diagnostics with either test modality. Only 2% (5/224) of the participants experienced symptoms (vomiting, anxiety, or claustrophobia) to a degree where they refused further examination with the MRC.

Another strength of this study is the validation of the examiner’s clinical conclusions by employing a secondary expert review of all recorded eye videos. The conclusions from this review were compared to the clinical conclusion in terms of agreement, which was satisfying. The agreement was not influenced by posterior and non-posterior subgroup analysis but tended to increase throughout the study period (Supplementary Table I). This tendency could either be explained by a learning curve in the examiner’s interpretation of the eye movements or by an overall improvement in the quality of the examinations and, as a direct result, a better quality of the recorded eye videos.

Limitations include the lack of examiner blinding. This means that the interpretation of the second diagnostic modality might have been influenced by the known results of the first diagnostic modality, introducing a potential confirmation bias. If confirmation bias were present, the second diagnostic modality would be expected to perform better than when the same modality was performed first, aligning with our observation that traditional MD showed a tendency of higher sensitivity when performed as the second diagnostic modality compared to when performed as the first diagnostic modality (Table 5). However, we did not observe a consistent pattern of confirmation bias, as MRC diagnostics showed no difference in sensitivity regardless of whether this diagnostic modality was performed as the first or the second diagnostic modality (Supplementary Table G). The reason why this tendency only applies to traditional MD remains unclear.

Another limitation is the risk of carryover effects, where the first diagnostic modality might influence the conditions for the second diagnostic modality. Head movements during the first diagnostic modality mobilized the otoconia, which were then unlikely to return to their original positions. Despite the fact that we tried to reduce the risk of carryover effects by (1) randomization of the order of diagnostic modalities and (2) inclusion of a 30-min washout period between the two diagnostic modalities, the specific location of otoconia were most likely not identical between the first and second diagnostic modality within the same participant.

### 4.4 Implications of results and future research

Our findings apply to an adult population presenting a typical BPPV case history. We compared BPPV diagnostics reflecting two distinct clinical settings: (1) ENT clinics (traditional MD with concomitant use of VNG goggles) and specialized tertiary clinics (MRC diagnostics with concomitant use of VNG goggles). Our study population primarily comprised patients referred by general practitioners, mirroring the typical patient demographics in ENT clinics with BPPV management. Our results offer valuable insights for ENTs when determining which BPPV patients may be managed at their clinic and which patients require referral to a specialized center for more accurate MRC diagnostics.

To better understand the impact of the concomitant use of VNG goggles with traditional MD, future studies should directly compare traditional MD performed with the naked eyes, Frenzel glasses, and both infrared and color VNG goggles. This will provide valuable insights regarding the baseline performance of traditional MD without any advanced equipment and provide knowledge on which type of technological assistance most effectively improves BPPV diagnostics. Research comparing different MRC devices is also warranted for a comprehensive evaluation of the diagnostic equipment available. Finally, determining the optimal order of positional testing in BPPV diagnostics remains critical for future investigations.

## 5 Conclusion

Traditional MD with concomitant use of VNG goggles remain reliable for the majority of patients suspected of having BPPV. However, MRC diagnostic is more accurate with specific BPPV cases. These include patients with (1) reduced cooperation during traditional MD, (2) patients with probable BPPV [negative (normal) traditional MD despite presenting a typical BPPV case history], and (3) patients referred from ENT clinics. MRC diagnostics may also be useful as a second opinion tool in patients with unsuccessful previous BPPV treatment attempts and in those with unclear BPPV subtype or SCC location.

While the concomitant use of VNG goggles with traditional MD might not improve the sensitivity compared to the concomitant use of Frenzel glasses with traditional MD, further research is needed to confirm this.

## Data Availability

The raw data supporting the conclusions of this article will be made available by the authors upon request, without undue reservation.
