# Supplementary material for: Is diagnostics of Benign Paroxysmal Positional Vertigo with a mechanical rotation chair superior to traditional manual diagnostics? A randomized controlled crossover study
Source: Front Neurol. 2024 Dec 6;15:1519837. doi: 10.3389/fneur.2024.1519837 (PMC11659124; doi:10.3389/fneur.2024.1519837)
Supplement: Supplementary file 1 [file Data_Sheet_1.pdf]

Supplementary Material

1 Supplementary Tables

Table A. Comparison of Included and Excluded Participants (n=279)

	**Included (n=224)**		**Excluded (n=55)**		
	n	*(%)*	*n*	*(%)*	*p-value*
Sex:					
Female, n (%)	152	(67.9)	39	(72.2)	0.54
Male, n (%)	72	(32.1)	15	(27.8)	-
Age, years (mean, ± SD)	58.2	± 15.8	60.9	± 15.4	0.25

The level of significance is p ≤ 0.05. Please note that no significant difference exists between the included and excluded participants, indicating no selection bias concerning sex and age.

Table B. BPPV Laterality

	**Right**		**Left**		
	n	*(%)*	*n*	*(%)*	*p-value*
Diagnostic modality					
Mechanical rotation chair(n=105)	53	(50.5)	41	(39.5)	0.00*
Traditional manual diagnostics (n=83)	47	(56.6)	29	(34.9)	0.00*
p-value	0.16		0.02*		

Significant p-values (p ≤ 0.05) are marked with an asterisk. Please note that both modalities have a significantly higher number of right-sided BPPV. Mechanical rotation chair diagnostics detected a significantly higher proportion of left-sided BPPV than traditional manual diagnostics.

Table C. Results of BPPV Diagnostics (n=215)

Mechanical Rotation Chair (columns) / **Traditional Manual Diagnostics** (rows)

	No BPPV	P-CAN dxt	P-CUP dxt	L-CAN dxt	L-CUP dxt	A-CAN dxt	A-CUP dxt	P-CAN sin	P-CUP sin	L-CAN sin	L-CUP sin	A-CAN sin	A-CUP sin	Unilateral multicanal	Bilateral single canal	Bilateral multicanal	Total
No BPPV	100	6	1	0	4	0	0	12	0	2	4	0	0	1	2	0	132
P-CAN dxt	4	26	0	0	0	0	0	0	1	0	0	0	0	5	0	0	36
P-CUP dxt	0	1	2	0	0	0	0	0	0	0	0	0	0	0	0	0	3
L-CAN dxt	0	0	0	0	0	0	0	0	0	0	0	0	0	0	0	0	0
L-CUP dxt	0	0	0	0	4	0	0	0	0	0	0	0	0	1	0	0	5
A-CAN dxt	0	0	0	0	0	1	0	0	0	0	0	0	0	0	0	0	1
A-CUP dxt	0	0	0	0	0	0	0	0	0	0	0	0	0	0	0	0	0
P-CAN sin	3	0	0	0	0	0	0	16	0	0	0	0	0	1	3	0	23
P-CUP sin	0	0	0	0	0	0	0	0	1	0	0	0	0	0	0	0	1
L-CAN sin	1	0	0	0	0	0	0	0	0	2	0	0	0	0	0	0	3
L-CUP sin	1	0	0	0	0	0	0	0	0	0	0	0	0	0	0	0	1
A-CAN sin	0	0	0	0	0	0	0	0	0	0	0	0	0	0	0	0	0
A-CUP sin	0	0	0	0	0	0	0	0	0	0	1	0	0	0	0	0	1
Unilateral multicanal	0	0	1	0	0	0	0	0	0	0	0	0	0	1	0	0	2
Bilateral single canal	1	0	0	0	0	0	0	0	0	0	0	0	0	0	5	0	6
Bilateral multicanal	0	0	0	0	0	0	0	0	0	0	0	0	0	0	1	0	1
Total	110	33	4	0	8	1	0	28	2	4	5	0	0	9	11	0	215

Total number of BPPV with traditional manual diagnostics: n=83

Total number of BPPV with mechanical rotation chair diagnostics: n= 105

Table showing the distribution of BPPVs between traditional manual diagnostics and mechanical rotation chair diagnostics.
CAN: Canalolithiasis; CUP: Cupulolithiasis; p: posterior; l: lateral; a: anterior.
Please note that the majority of BPPV cases are placed in the diagonal midline, indicating agreement between the two diagnostic modalities.

Table D. Results of BPPV Diagnostics

Sufficient cooperation during traditional manual diagnostics (n=175)

		**MRC**			p-value	
		BPPV	No BPPV	Total	0.02*	
**MD**	BPPV	58	7	65 (37.1)		
	No BPPV	19	91	110 (62.9)	Accuracy	(95% CI)
	Total	77 (44.0)	98 (56.0)	175 (100)	73.5%	(67.6-79.4)

Impaired but acceptable cooperation during traditional manual diagnostics (n=40)

		**MRC**			*p-value*	
		BPPV	No BPPV	Total	0.01*	
**MD**	BPPV	15	3	18 (45.0)		
	No BPPV	13	9	22 (55.0)	*Accuracy*	*(95% CI)*
	Total	28 (70.0)	12 (30.0)	40 (100)	14.4%	(9.7-19.1)

Referred by a general practitioner (n=184)

		**MRC**			*p-value*	
		BPPV	No BPPV	Total	0.00*	
**MD**	BPPV	61	6	67 (36.4)		
	No BPPV	25	92	117 (63.6)	*Accuracy*	*(95% CI)*
	Total	86 (46.7)	98 (53.2)	184 (100)	77.2%	(71.6-82.8)

Referred by an otorhinolaryngologist clinic (n=31)

		**MRC**			*p-value*	
		BPPV	No BPPV	Total	0.37	
**MD**	BPPV	12	4	16 (51.6)		
	No BPPV	7	8	15 (48.4)	*Accuracy*	*(95% CI)*
	Total	19 (61.3)	12 (38.7)	31 (100)	10.7%	(6.6-14.8)

MRC first – MD second (n=109)

		**MRC**			*p-value*	
		BPPV	No BPPV	Total	0.09	
**MD**	BPPV	36	5	41 (37.6)		
	No BPPV	12	56	68 (62.4)	*Accuracy*	*(95% CI)*
	Total	48 (44.0)	61 (56.0)	109 (100)	43.8%	(36.2-49.4)

MD first – MRC second (n=106)

		**MRC**			*p-value*	
		BPPV	No BPPV	Total	0.00*	
**MD**	BPPV	37	5	42 (39.6)		
	No BPPV	20	44	64 (60.4)	*Accuracy*	*(95% CI)*
	Total	57	49	106	37.7%	(31.2-44.2)

	(53.8)	(46.2)	(100)

MRC: mechanical rotation chair; MD: traditional manual diagnostics.
Results are expressed as n (%). Percentages refer to the total sample in each group.
Significant p-values (p-value ≤ 0.05) are marked with an asterisk. Please note that MRC diagnostics was significantly more sensitive in diagnosing BPPV in all groups except those referred by ENTs and those who underwent examination with an MRC first.

Table E. Comparison of BPPV by Referrals

	**General Practitioner (n=184)**		**ENT clinics (n=31)**		
	n	*(%)*	*n*	*(%)*	*p-value*
**Mechanical rotation chair**					
BPPV total	86	(46.7)	19	(61.3)	0.13
BPPV localization:	*n=86*		*n=19*		
Single canal BPPV	71	(82.6)	14	(73.7)	0.49
Posterior BPPV	60	(69.8)	7	(36.8)	0.25
Lateral BPPV	10	(11.6)	7	(36.8)	0.01*
Anterior BPPV	1	(1.2)	0	(0.0)	-
Multicanal BPPV (≥2 SCC):	15	(17.4)	5	(26.3)	0.18
Non-posterior BPPV	26	(30.2)	12	(63.2)	0.00*
**Traditional manual diagnostics**					
BPPV total	67	(36.4)	16	(51.6)	0.11
BPPV localization:	*n=67*		*n=16*		
Single canal BPPV	60	(89.6)	14	(87.5)	0.18
Posterior BPPV	53	(79.1)	10	(62.5)	0.70
Lateral BPPV	6	(9.0)	3	(18.8)	0.12
Anterior BPPV	1	(1.5)	1	(6.3)	0.27
Multicanal BPPV (≥2 SCC):	7	(10.5)	2	(12.5)	0.62
Non-posterior BPPV	14	(20.9)	6	(37.5)	0.05

ENT: otorhinolaryngologist; CAN: canalolithiasis; CUP: cupulolithiasis; SCC: semicircular canal.
Significant p-values (p ≤ 0.05) are marked with an asterisk. Please note that significantly more participants were diagnosed with non-posterior BPPV with a mechanical rotation chair when referred by ENTs.

Table F. Comparison of BPPV in First and Second Examination (n=215)

	First examination		Second examination		
	n	*(%)*	*n*	*(%)*	*p-value*
**Mechanical rotation chair diagnostics**	*n=109*		*n=106*		
BPPV total	48	(44.0)	57	(53.8)	0.15
BPPV subtype and localization:	*n=48*		*n=57*		
Single canal BPPV	37	(77.1)	48	(84.2)	0.09
Posterior BPPV total	28	(58.3)	39	(68.4)	0.08
Lateral BPPV total	8	(16.7)	9	(15.8)	0.75
Anterior BPPV total	1	(2.08)	0	(0.0)	-
Multicanal BPPV (≥2 SCC):	11	(22.9)	9	(15.8)	0.69
Non-posterior BPPV	20	(41.7)	18	(31.6)	0.79
**Traditional manual diagnostics**	*n=106*		*n=109*		
BPPV total	42	(39.6)	41	(37.6)	0.76
BPPV subtype and localization:	*n=42*		*n=41*		
Single canal BPPV	41	(97.6)	41	(80.5)	0.20
Posterior BPPV total	35	(83.3)	28	(68.3)	0.24
Lateral BPPV total	5	(11.9)	4	(9.8)	0.75
Anterior BPPV total	1	(2.4)	1	(2.4)	-
Multicanal BPPV (≥2 SCC):	1	(2.4)	8	(19.5)	0.04*
Non-posterior BPPV	7	(16.7)	13	(31.7)	0.18

CAN: canalolithiasis; CUP: cupulolithiasis; SCC: semicircular canal
Significant p-values ($p \leq 0.05$) are marked with an asterisk. Please note that there are no significant differences between findings (except multicanal BPPV with traditional manual diagnostics, all represented by bilateral posterior BPPV) in the modalities when performed first or second in the study.

Table G. Diagnostic Profile of Mechanical Rotation Chair (n=215)

	Mechanical rotation chair diagnostics compared to traditional manual diagnostics							
	Sensitivity		Specificity		PPV		NPV	
	%	*(95% CI)*	*%*	*(95% CI)*	*%*	*(95% CI)*	*%*	*(95% CI)*
All BPPV	88.0	(79.0-94.1)	75.8	(67.5-82.8)	69.5	(59.8-78.1)	90.9	(83.9-95.6)
Posterior BPPV	74.6	(62.1-95.8)	86.8	(80.4-91.8)	70.1	(57.7-80.7)	89.2	(83.0-93.7)
Non-posterior BPPV	80.0	(56.3-94.3)	88.7	(93.4-92.8)	42.1	(26.3-59.2)	97.7	(94.3-99.4)
Referred by:								
General practitioner (n=184)	91.0	(81.5-96.6)	78.6	(70.1-85.7)	70.9	(60.1-80.2)	93.9	(87.1-97.7)
ENT clinics (n=31)	75.0	(47.6-92.7)	53.3	(26.6-78.7)	63.2	(38.4-83.7)	66.7	(34.9-90.1)
When MRC was performed:								
First	87.8	(73.8-95.9)	82.4	(71.2-90.5)	75.0	(60.4-86.4)	91.8	(81.9-97.3)
Second	88.1	(74.4-96.0)	68.8	(55 9-79.8)	64.9	(51.1-77.1)	89.8	(77 8-96.6)

MRC: mechanical rotation chair (index test); MD: traditional manual diagnostics (reference test); PPV: positive predictive value; NPV: negative predictive value; ENT: otorhinolaryngologist

Table H. Comparison of Study Periods (n=215)

	First study period (n=108)		Last study period (n=107)		
	n	*(%)*	*n*	*(%)*	*p-value*
Diagnosed BPPV total	60	(55.6)	55	(51.4)	0.54
Diagnosed BPPV with MRC	54	(50.0)	51	(47.7)	0.73
Diagnosed BPPV with MD	43	(39.8)	40	(37.4)	0.71
BPPV with the use of MRC					
	n=54		*n=51*		
Posterior BPPV	36	(66.7)	31	(60.8)	0.49
Non-posterior BPPV	18	(33.3)	20	(39.2)	0.70
BPPV with traditional MD					
	n=43		*n=40*		
Posterior BPPV	33	(76.7)	30	(75.0)	0.69
Non-posterior BPPV	10	(23.3)	10	(25.0)	0.98

MRC: mechanical rotation chair; MD: manual diagnostics
The level of significance is $p \leq 0.05$. Please note that there was no significant difference in the findings between the study periods, indicating that the examiner's learning curve did not influence the results.

Table I. Second Review of Eye Recordings

	Overall agreement		Cohen's kappa	
	%	*(95% CI)*		*(95% CI)*
Examination with the use of MRC				
BPPV or no BPPV	89.8	(85.7-93.8)	0.79	(0.66-0.93)
Posterior BPPV	94.0	(90.8-97.1)	0.86	(0.72-0.99)
Non-posterior BPPV	94.9	(91.9-97.8)	0.81	(0.67-0.94)
Study periods				
First study period (n=108)	84.3	(77.4-91.1)	0.69	(0.51-0.87)
Second study period (n=107)	95.3	(91.3-99.3)	0.91	(0.72-1.10)
Examination with traditional MD				
BPPV or no BPPV	92.5	(88.5-95.7)	0.84	(0.70-0.96)
Posterior BPPV	93.5	(90.1-96.8)	0.84	(0.71-0.97)
Non-posterior BPPV	96.3	(93.7-98.8)	0.75	(0.61-0.88)
Study periods				
First study period (n=108)	87.0	(80.7-93.4)	0.74	(0.55-0.92)
Second study period (n=107)	97.2	(94.1-1.00)	0.94	(0.75-1.13)

The second review was performed by an expert blinded to the examiner's clinical conclusion.
MRC: mechanical rotation chair; MD: manual diagnostics.
Please note the tendency of lower Cohen's kappa with diagnostics of non-posterior BPPV. Also, note the higher Cohen's kappa in participants from the second study period compared to the first. All p-values for Cohen's kappa are ≤0.05, indicating that the agreement between the modalities is beyond chance.
